# Lifestyle factors associated with overweight and obesity among Saudi adolescents

**DOI:** 10.1186/1471-2458-12-354

**Published:** 2012-05-16

**Authors:** Hazzaa M Al-Hazzaa, Nada A Abahussain, Hana I Al-Sobayel, Dina M Qahwaji, Abdulrahman O Musaiger

**Affiliations:** 1 Director of Exercise Physiology Laboratory, College of Education, King Saud University, P. O. Box. 2458, Riyadh, 11451, Saudi Arabia; 2 Scientific Boards, Obesity Research Chair, King Saud University, Riyadh, Saudi Arabia; 3 Director of School Health, Ministry of Education, Eastern Province, Saudi Arabia; 4 Department of Rehabilitation Sciences, College of Applied Medical Sciences, King Saud University, Riyadh, Saudi Arabia; 5 Department of Clinical Nutrition, College of Applied Medical Sciences, King Abdulaziz University, Jeddah, Saudi Arabia; 6 Arab Center for Nutrition, Manama, Bahrain, and Nutrition and Health Studies Unit, Deanship of Scientific Research, University of Bahrain, Manama, Bahrain

**Keywords:** Adolescents, Dietary habits, Lifestyle, Overweight, Obesity, Physical activity, Saudi Arabia, Sedentary behaviors

## Abstract

**Background:**

A better understanding of the relationships between obesity and lifestyle factors is necessary for effective prevention and management of obesity in youth. Therefore, the objective of this study was to evaluate the associations between obesity measures and several lifestyle factors, including physical activity, sedentary behaviors and dietary habits among Saudi adolescents aged 14–19 years.

**Methods:**

This was a school-based cross-sectional study that was conducted in three cities in Saudi Arabia (Al-Khobar, Jeddah and Riyadh). The participants were 2906 secondary school males (1400) and females (1506) aged 14–19 years, who were randomly selected using a multistage stratified cluster sampling technique. Measurements included weight, height, body mass index (BMI), waist circumference, waist/height ratio (WHtR), screen time (television viewing, video games and computer use), physical activity (determined using a validated questionnaire), and dietary habits (intake frequency per week). Logistic regression was used to examine the associations between obesity and lifestyle factors.

**Results:**

Compared with non-obese, obese males and females were significantly less active, especially in terms of vigorous activity, had less favorable dietary habits (e.g., lower intake of breakfast, fruits and milk), but had lower intake of sugar-sweetened drinks and sweets/chocolates. Logistic regression analysis showed that overweight/obesity (based on BMI categories) or abdominal obesity (based on WHtR categories) were significantly and inversely associated with vigorous physical activity levels (aOR for high level = 0.69, 95% CI 0.41–0.92 for BMI and 0.63, 95% CI 0.45–0.89 for WHtR) and frequency of breakfast (aOR for < 3 days/week = 1.44; 95% CI 1.20–1.71 for BMI and 1.47; 95% CI 1.22–1.76 for WHtR) and vegetable (aOR for < 3 days/week = 1.29; 95% CI 1.03–1.59 for WHtR) intakes, and consumption of sugar-sweetened beverages (aOR for < 3 days/week = 1.32; 95% CI 1.08–1.62 for BMI and 1.42; 95% CI 1.16–1.75 for WHtR).

**Conclusions:**

The present study identified several lifestyle factors associated with obesity that may represent valid targets for the prevention and management of obesity among Saudi adolescents. Primary prevention of obesity by promoting active lifestyles and healthy diets should be a national public health priority.

## Background

In recent years, obesity among children and adolescents has emerged as a global epidemic [1] and is becoming a serious public health problem in the Eastern Mediterranean region [2-4]. In Saudi Arabia, a country that has experienced marked nutritional changes and rapid urbanization in recent decades, it was estimated that 26.6% and 10.6% of adolescents aged 13–18 years are overweight or obese, respectively [5]. Furthermore, evidence from serial cross-sectional assessments of body mass index (BMI) or percent body fat that were carried out in Saudi children and adolescents have confirmed the rising trend in obesity over the last two decades [6-8].

It is believed that the rise of obesity in developing countries is likely to create a tremendous public health burden [9], because obesity in children and adolescents is strongly associated with many comorbidities [10-12]. Metabolic complications associated with obesity in childhood greatly increase the risk for type 2 diabetes and early cardiovascular disease [13]. Moreover, obesity in adolescence was shown to track to adulthood [14,15]. Aside from overall obesity, abdominal obesity has also been linked to increased cardiometabolic risk in children and adolescents [16,17]. The use of the waist to height ratio (WHtR) to determine abdominal obesity in children is simple, sensitive, and seems to better predict cardiometabolic disease risk in children and adolescents than does BMI [18,19]. Therefore, considering the long-term adverse effects of childhood obesity, early identification of high BMI and the prevention of excess weight gain are strongly advocated [10].

Current evidence indicates that obesity is a multifactorial condition influenced by many variables, including genetic, demographic and lifestyle factors [1,10]. Genetic and demographic variables such as family history of obesity, age, ethnicity and sex cannot be modified. However, obesity-associated lifestyle factors are often modifiable. In fact, previous research has shown that childhood obesity is associated with many lifestyle factors, including sedentary behaviors [20,21], physical inactivity [22,23] and unhealthy dietary choices [24-26]. However, not all of the studies showed associations between childhood obesity and some lifestyle factors, such as the consumption of soda beverages [26] or computer use [27].

Information on lifestyle factors associated with obesity in adolescents in Saudi Arabia are currently limited [28-31], and the available data indicated that unhealthy dietary choices and inactivity were generally correlated with BMI in Saudi children and adolescents [28-31]. However, local studies using a representative sample and validated instruments to assess lifestyle factors are particularly scarce. This is quite surprising, considering the fact that Saudi Arabia has experienced enormous lifestyle changes in recent decades along with the rise in childhood obesity [6-8]. Therefore, a better understanding of the relationships between obesity and lifestyle factors is necessary for effective prevention and management of obesity in youth. Accordingly, the objective of the present study was to evaluate the associations between overweight, obesity and abdominal obesity and several lifestyle factors, including physical activity, sedentary behaviors and dietary habits among Saudi adolescents aged 14–19 years, using representative samples drawn from three major cities in Saudi Arabia.

## Methods

### Study sample

The present study is part of the Arab Teens Lifestyle Study (ATLS), a school-based cross-sectional multicenter collaborative project [32]. The participants are adolescent males and females enrolled across secondary schools in three major cities in Saudi Arabia; Riyadh is the capitol of Saudi Arabia and is located in the central region, Jeddah is the second largest city and is located on the shore of the Red Sea in western Saudi Arabia, and Al-Khobar is a modern city located in the eastern province of Saudi Arabia.

The minimum sample size in each city was determined so that the sample proportion would be within ± 0.05 of the population proportion with a 95% confidence level. The population proportion has been assumed to be 0.50, as this magnitude yield the maximum possible sample size required. For example, in the city of Riyadh, where the population of male students in the public and private secondary schools was about 75000 at that time, the minimum needed sample size for male students was 382. The sample size was then increased by 10–15% to account for missing data.

A multistage stratified cluster random sampling technique was used to select the sample. In the first stage, a systematic random sampling procedure was used to select the schools. The schools were stratified into boys and girls secondary schools, with further stratification into public and private schools. The selection of private/public schools was proportional to population size. Four schools (two each from the boys and girls schools) were selected from each of the four geographical areas in each city (i.e., east, north, south and west). At the second stage, classes were selected at each grade (level) using a simple random sampling design. In this way, one class was randomly selected in each of the three grades (grades 10, 11 and 12) in each secondary school. Thus, we selected at least 24 classes in each city (12 each from the boys and girls schools). All students in the selected classes, who were free from any physical deformity, were invited to participate in the study. Because of differences in class size between cities and between private and public schools, the sample sizes for the participating cities differ. In addition, some cities have more dispersed schools, which required selecting more schools to achieve a better representation.

The data were collected during the months of October and November of 2009. The study protocol and procedures were approved by the Research Center at King Saud University as well as by the General Directorate of School Education in each city. We also obtained schools and parental consents as well as students’ approval for conducting the survey. The total sample size consisted of 2906 adolescents (1400 males and 1506 females).

### Anthropometric measurements

Anthropometric variables included body weight, height and waist circumference (WC). Measurements were performed in the morning by trained researchers using standardized procedures. Body weight was measured to the nearest 100 g, with minimal clothing and without shoes, using a calibrated portable scale. Height was measured to the nearest cm with the subject in the full standing position without shoes using calibrated portable measuring rod. Body mass index (BMI) was calculated as body weight in kg divided by height squared in meters. The International Obesity Task Force (IOTF) age- and sex-specific BMI reference values were used to define overweight and obesity in adolescents aged 14–17 years [33]. For participants aged ≥18 years, we used the cut-off points for adults (overweight, 25–29.9 kg/m^2^; obesity ≥30 kg/m^2^). WC was measured horizontally at navel level and at the end of gentle expiration to the nearest 0.1 cm using a non-stretchable measuring tape. Waist height ratio (WHtR) was calculated as the ratio between WC in cm and height in cm. A WHtR cut-off point of 0.50 was used to define abdominal obesity in males and females [18,19].

### ATLS research instrument

The ATLS research instrument [32,34] that was used to record lifestyle information consisted of 47 items. Of these, the first five items were essential, and were age, weight, height, waist circumference and student’s level of study. Items 6–34 comprised the physical activity questionnaire, items 35–37 recorded sedentary activities and items 38–47 focused on dietary habits. To ensure accurate and consistent measurements throughout this multicenter project, all data collection centers followed a standardized protocol. Written instructions were provided to the researchers on sampling procedures, weight, height and waist circumference measurements, and how to conduct the questionnaire.

### Physical activity assessment

Because of the nature and diversity of the ATLS project, a self-reported questionnaire was used to assess the level of physical activity of the participants. The original questionnaire was previously shown to have a high reliability (intraclass coefficient 0.85; 95% confidence interval [CI] 0.70–0.93) and acceptable validity (*r* = 0.30; *p* < 0.05) against pedometer-assessed activity in a convenient sample of young males aged 15–25 years [35,36]. The ATLS physical activity questionnaire used in our study was also validated against pedometer-assessed activity in females and males aged 14–19 years [37], and had an acceptable validity coefficient (*r* = 0.37, *p* < 0.001).

The participants completed the ATLS questionnaire in their classrooms under supervision of their teachers and least one research assistant. The physical activity part of the questionnaire was designed to collect information on the frequency, duration and intensity of light-, moderate- and vigorous-intensity physical activities during a typical (usual) week. The physical activity questionnaire covers several domains, including transport, the household, fitness and sporting activities. Physical activities were assigned metabolic-equivalent (MET) values based on the compendium of physical activity [38] and the compendium of physical activity for youth [39]. Moderate-intensity physical activities include normal pace walking, brisk walking, recreational swimming, household activities, and recreational sports such as volleyball, badminton and table tennis. Moderate-intensity recreational sports were assigned an average MET value equivalent to 4 METs. Household activities were given an average MET value of 3. Slow walking, normal pace walking and brisk walking were assigned MET values of 2.8, 3.5 and 4.5 METs respectively, based on the modified MET values in the compendium of physical activity for youth [39]. Vigorous-intensity physical activities and sports included stair climbing, jogging, running, cycling, self-defense, weight training, soccer, basketball, handball, and singles tennis. Vigorous-intensity sports were assigned an average MET value of 8. To measure the participants’ levels of physical activity, we used the total METs-min per week and the METs-min per week spent in each of the moderate- and vigorous-intensity physical activity. For physical activity cut-off values, we used three categories (low, medium and high activity) based on tertiles of total METs-min per week, METs-min per week from vigorous-intensity physical activity, and METs-min per week from moderate–intensity physical activity.

### Sedentary behaviors

The questions on sedentary behaviors followed the physical activity questions, and were designed to asses typical time spent per day on sedentary activities, including television (TV) viewing, video games, and computer and internet use. Participants were asked to state their typical time (hours) spent on these activities without differentiating between weekdays and weekend. For total screen viewing time cut-off values, we used the American Academy of Pediatric guidelines of a maximum of 2 h per day [40].

### Eating habits

The ATLS questionnaire also included 10 specific questions designed to assess the frequency of certain dietary habits during a typical (usual) week. The questions asked the participants to state how many times per week they consume breakfast, sugar-sweetened drinks (including soft drinks), vegetables (cooked and uncooked), fruits, milk and dairy products, donuts/cakes, sweets and chocolates, energy drinks and fast foods. The fast foods in this regard included some examples from Western fast foods and Arabic fast foods, such as *shawarma* (grilled meat or chicken in pita bread with some salad). The questions covered some healthy and unhealthy dietary habits. The student was given a choice of answers, ranging from zero intake (never) to a maximum intake of 7 days per week (every day). We categorized the dietary habits into three levels of intake: ≥5 days per week, 3–4 days per week and <3 days per week.

### Data and statistical analysis

Data at each center were checked and entered into a computer using standardized entry codes written on an SPSS data file. The entered data were then sent to a central processing center (Riyadh). At the central processing center, all data were checked for outliers or incorrect/illogical entries. Data were then analyzed using SPSS version 15 (SPSS, Inc, Chicago, IL, USA). Descriptive statistics are presented as means ± standard deviations (SD) or proportions. Data that were not normally distributed, such as physical activity scores in METs-min per week, were log transformed before performing analysis of variance (ANOVA). Differences in anthropometric measurements between regions were tested separately for each of the males and females using one-way ANOVA. The proportions of Saudi males and females who exceeded specific cut-off values for sedentary behaviors, physical activity and dietary habits were calculated. We also used two-way analysis of covariance (ANCOVA; sex **×** BMI category, and sex **×** WHtR category) while controlling for the effects of age to test for differences in lifestyle variables across sex (males and females) and obesity indicators. Finally, we performed multinomial logistic regression to examine the independent associations between sex, school type and lifestyle factors with each of dependent measures of obesity (overweight/obesity versus normal weight and above versus below 50% of WHtR), which were entered separately. In preliminary logistic regression models, we adjusted our analysis for age, location, sex and public/private schools. However, this did not materially change the observed associations and was thus excluded. Adjusted-for-age odds ratios (aORs) and 95% CIs were calculated for each independent variable. In these models, several parameters (namely the consumption of fast foods, French fries, cakes/donuts, sweets and energy drinks) were set to zero because they were redundant due to high co-linearity with other independent variables. The level of significance was set at *p <* 0.05.

## Results

The descriptive characteristics of the participants are shown in Table 1. The percentage of females in the sample slightly exceeded that of males (51.8% versus 48.2%). The mean ages of males and females were 16.7 and 16.5 years, respectively. There were significant (*p* < 0.05) differences between males and females for age, weight, height, BMI, WC and the combined prevalence of overweight and obesity.

**Table 1:** **Subject characteristics (n = 2906**)

**Variable**	**Male**	**Female**
Number of participants	1400	1506
Age (yr)	16.7 ± 1.1	16.5 ± 1.1 ^**a**^
Weight (kg)	70.0 ± 20.5	57.9 ± 15.5 ^**a**^
Height (cm)	168.4 ± 7.2	156.7 ± 5.9 ^**a**^
BMI (kg/m^**2**^)	24.6 ± 6.7	23.6 ± 6.1 ^**a**^
WC (cm)	79.7 ± 15.4	74.2 ±13.1 ^**a**^
Overweight and obesity (%)	43.6	34.8 ^**b**^

Data are means ± standard deviation.

^**a**^*p* < 0.05 vs males (*t*-test for independent samples).

^**b**^*p* < 0.01 vs males (*t*-test for proportions).

BMI = body mass index; WC = waist circumference.

Table 2 shows the mean ± SD values for anthropometric and lifestyle-related variables stratified by sex and BMI category. Two-way ANCOVA tests revealed that there were significant (*p* < 0.05) interactions between sex and BMI category in body weight, WC, total METs min per week, METs-min of vigorous-intensity physical activity and the intake of milk/dairy products and sweets. Age, as a covariate, exerted significant (*p* < 0.05) effects over the majority of the selected anthropometric and lifestyle variables. The interaction sex × BMI category was significant for several of the variables shown in Table 2. Compared with males, Saudi females, were significantly (*p* < 0.05) more sedentary, much less active, especially in terms of vigorous activity, and consumed breakfast, fruits, milk/dairy products, sugar-sweetened drinks, fast foods and energy drinks on fewer days per week. However, the weekly intake of French fries/potato chips, cakes/donuts, and sweets/chocolates were significantly (*p* < 0.05) higher in females than in males.

**Table 2:** Lifestyle-related variables among Saudi adolescents stratified by sex and BMI categories

**Variable**	**Male**			**Female**		
	**Normal**	**Overweight**	**Obese**	**Normal**	**Overweight**	**Obese**
Age (years) ^***b***^	16.8 ± 1.1	16.7 ± 1.2	16.8 ± 1.1	16.5 ± 1.0	16.5 ± 1.1	16.6 ± 1.1
Weight (kg) ^***a, b, c, d***^	56.1 ± 8.5	75.2 ± 7.4	98.3 ± 16.1	49.6 ± 7.1	65.4 ± 6.3	85.7 ± 15.8
Height (cm) ^***a,b***^	168.0 ± 7.4	168.4 ± 6.6	169.1 ± 7.1	156.6 ± 5.7	156.7 ± 6.4	168.5 ± 6.0
BMI (kg/m^**2**^) ^***a, b, c***^	19.8 ± 2.4	26.5 ± 1.5	34.3 ± 4.8	20.2 ± 2.5	26.6 ± 1.5	34.9 ± 6.0
Waist Circumference (cm) ^***a, b, c, d***^	69.8 ± 7.7	83.7 ± 8.5	99.8 ± 11.9	68.1 ± 7.2	79.4 ± 7.4	94.7 ± 16.4
Waist-height ratio ^***a, b, c***^	0.415 ± 0.04	0.497 ± 0.05	0.590 ± 0.07	0.435 ± 0.04	0.507 ± 0.05	0.605 ± 0.10
Screen time (TV viewing and computer use) hour/day ^***b***^	5.25 ± 3.2	5.29 ± 3.3	5.5 ± 3.4	6.60 ± 3.6	6.53 ± 3.3	6.56 ± 3.9
METs-min/week of Moderate –intensity physical activity ^***b***^	1016.3 ± 1185.9	1044.9 ± 1097.9	1054.4 ± 1166.5	721.3 ± 833.6	826.1 ± 909.4	791.4 ± 1005.6
METs-min/week of Vigorous –intensity physical activity ^***a, b, c, d***^	2457.1 ± 2761.1	2067.1 ± 2292.5	1757.1 ± 2130.2	564.7 ± 910.6	574.9 ± 924.9	482.3 ± 625.7
Total METs-min/week ^***a, b, c, d***^	3365.4 ± 3485.8	3022.4 ± 2869.2	2699.9 ± 2793.7	1194.0 ± 1413.9	1280.3 ± 1512.3	1202.4 ± 1363.8
Breakfast consumption (frequency/week) ^***a, b, c***^	3.94 ± 2.7	3.52 ± 2.7	3.35 ± 2.7	3.46 ± 2.6	3.08 ± 2.5	3.79 ± 2.6
Vegetables Consumption (frequency/week) ^***a***^	3.77 ± 2.4	3.67 ± 2.4	3.62 ± 2.4	3.62 ± 2.4	3.67 ± 2.4	3.65 ± 2.4
Fruits Consumption (frequency/week) ^***a, b***^	3.27 ± 2.2	3.40 ± 2.4	3.16 ± 2.3	2.47 ± 2.1	2.71 ± 2.2	2.51 ± 2.1
Milk/diary products intake (frequency/week) ^***a, b, c***^	4.45 ± 2.4	4.21 ± 2.5	4.07 ± 2.5	3.71 ± 2.5	3.77 ± 2.5	3.26 ± 2.6
Sugar-sweetened drinks (frequency/week) ^***b, c, d***^	4.82 ± 2.3	4.52 ± 2.5	4.76 ± 2.3	4.36 ± 2.3	4.19 ± 2.3	3.78 ± 2.3
Fast foods (frequency/week) ^***b***^	2.93 ± 1.9	2.82 ± 2.1	2.89 ± 2.0	2.68 ± 1.8	2.69 ± 2.0	2.89 ± 2.0
French fries/potato chips (frequency/week) ^***b, c***^	2.51 ± 2.1	2.39 ± 2.1	2.49 ± 2.1	2.99 ± 2.0	2.55 ± 2.0	2.60 ± 2.0
Cake/donuts (frequency/week) ^***c***^	2.55 ± 2.1	2.29 ± 2.0	2.39 ± 2.0	2.89 ± 2.1	2.52 ± 1.9	2.32 ± 1.9
Sweets (frequency/week) ^***b, c, d***^	3.19 ± 2.3	2.85 ± 2.3	2.84 ± 2.3	4.19 ± 2.3	3.67 ± 2.2	3.18 ± 2.1
Energy drinks (frequency/week) ^***b***^	1.51 ± 2.1	1.61 ± 2.2	1.59 ± 2.2	0.89 ± 1.7	0.91 ± 1.7	0.82 ± 1.6

Data are means and standards deviations and were analyzed by ANCOVA controlling for age.

^**a**^*p* < 0.05 for the effect of age.

^**b**^*p* < 0.05 for the main effect of sex.

^**c**^*p* < 0.05 for the main effect of BMI category.

^**d**^*p* < 0.05 for the effect of the interaction sex × BMI category.

Obese males and females appeared to be much less active than their non-obese counterparts, particularly in terms of vigorous activity. They also had less favorable dietary habits, include less frequent intake of breakfast, fruits and milk, but had lower intake of sugar-sweetened drinks, and sweets/chocolates compared with non-obese.

The means ± SD values of anthropometric and lifestyle-related variables stratified by sex and WHtR cut-off values are shown in Table 3. Again, ANCOVA revealed that many of the variables exhibited significant (*p* < 0.05) interactions (sex × WHtR cut-offs), although the intake of sweets was the only dietary habit that showed this interaction. Age was also a significant cofactor in most of the studied variables. Similar to the results in Table 2, there were marked male/female patterns in terms of lifestyle factors; for example, females were more sedentary and less active, and significantly (*p* < 0.05) less frequently consumed breakfast, fruits, milk, sugar-sweetened drinks, and energy drinks, but significantly more frequently consumed French fries/potato chips, cakes/donuts, and sweets/chocolates. In addition, males and females with high WHtR tended to be report lower total and vigorous-intensity physical activity as well as less healthy dietary choices, including less frequent consumption of breakfast and milk. However, those with higher level of WHtR seemed to less frequently consume French fries, sugar-sweetened beverages, cake/donuts, and sweets/chocolates.

**Table 3:** Lifestyle-related variables among Saudi adolescents stratified by sex and waist to height ratio (WHtR) categories

**Variable**	**Male**			**Female**				
	**WHtR < 0.5**	**WHtR** ≥ **0.5**	**WHtR < 0.5**	**WHtR** ≥ **0.5**				
Age (years) ^**b**^	16.7 ± 1.1	16.8 ± 1.1	16.5 ± 1.0	16.5 ± 1.1
Weight (kg) ^**a, b, c, d**^	58.8 ± 10.9	89.8 ± 18.5	51.7 ± 9.8	72.2 ± 16.7
Height (cm) ^**a, b, d**^	168.1 ± 7.3	168.8 ± 6.8	156.9 ± 5.7	156.0 ± 6.3
BMI (kg/m^**2**^) ^**a,b, c, d**^	20.8 ± 3.4	31.4 ± 5.7	20.9 ± 3.7	29.6 ± 6.3
Waist circumference (cm) ^**b, c, d**^	70.3 ± 7.5	96.5 ± 10.9	67.7 ± 6.3	88.9 ± 12.8
Waist to height ratio ^**b, c, d**^	0.418 ± 0.04	0.572 ± 0.06	0.432 ± 0.04	0.570 ± 0.08
Screen time (television viewing and computer use) hour/day ^**b**^	5.32 ± 3.3	5.32 ± 3.2	6.59 ± 3.5	6.56 ± 3.7
METs-min/week of moderate–intensity physical activity ^**b**^	1005.1 ± 1086.1	991.9 ± 1086.2	734.3 ± 861.7	799.1 ± 912.2
METs-min/week of vigorous-intensity physical activity ^**a, b, c, d**^	2366.5 ± 2454.7	1763.9 ± 2066.5	573.7 ± 930.2	515.1 ± 753.7
Total METs-min/week ^**a, b, c, d**^	3266.2 ± 3034.5	2659.1 ± 2670.6	1210.3 ± 1447.5	1224.7 ± 1387.9
Breakfast consumption ^**a, b, c**^ (frequency/week)	3.89 ± 2.7	3.39 ± 2.7	3.47 ± 2.6	2.85 ± 2.5
Vegetable consumption (frequency/week) ^**a**^	3.78 ± 2.4	3.58 ± 2.3	3.68 ± 2.4	3.53 ± 2.3
Fruit consumption (frequency/week) ^**a,b**^	3.30 ± 2.3	3.19 ± 2.2	2.52 ± 2.1	2.53 ± 2.2
Milk/diary product intake ^**a, b**, c^ (frequency/week)	4.47 ± 2.4	4.92 ± 2.5	3.73 ± 2.5	3.49 ± 2.5
Sugar-sweetened drinks ^**a, b, c**^ (frequency/week)	4.80 ± 2.4	4.65 ± 2.4	4.34 ± 2.4	4.02 ± 2.3
Fast food intake ^**b**^ (frequency/week)	2.94 ± 1.9	2.82 ± 2.0	2.66 ± 1.8	2.54 ± 1.9
French fries/potato chip intake ^b, c^ (frequency/week)	2.49 ± 2.1	2.47 ± 2.0	2.93 ± 2.0	2.61 ± 2.0
Cake/donut intake ^**b, c**^ (frequency/week)	2.52 ± 2.1	2.35 ± 2.0	2.84 ± 2.1	2.51 ± 1.9
Sweet intake (frequency/week) ^**b, c, d**^	3.14 ± 2.3	2.87 ± 2.3	4.14 ± 2.3	3.49 ± 2.2
Energy drink intake ^**b**^ (frequency/week)	1.49 ± 2.1	1.65 ± 2.3	0.89 ± 1.7	0.88 ± 1.7

Data are means and standards deviations, and were analyzed by ANCOVA controlling for age.

^**a**^*p* < 0.05 for the effect of age.

^**b**^*p* < 0.05 for the main effect of sex.

^**c**^*p* < 0.05 for the main effect of WHtR category.

^**d**^*p* < 0.05 for the effect of the interaction sex × WHtR category.

Table 4 shows the results of the multiple logistic regression analyses. The regression coefficients (β) showed that the following factors were significantly associated with overweight and obesity: being a male (aOR 1.73; 95% CI 1.44–2.07), being in a private school (aOR 1.50; 95% CI 1.26–1.78), consuming breakfast on <3 days per week (aOR 1.44; 95% CI 1.20–1.71) and consuming sugar-sweetened drinks on 3–4 days per week (aOR 1.27; 95% CI 1.05–1.53) or <3 days per week (aOR 1.32; 95% CI 1.08–1.62), while vigorous physical activity was associated with reduced risk of being overweight or obesity (medium level: aOR 0.72, 95% CI 0.49–0.96; high level: aOR 0.69, 95% CI 0.41–0.92). For central obesity (i.e., WHtR > 0.50), the following factors were associated with increased risk of abdominal obesity: being a male (aOR 1.52; 95% CI 1.26–1.84), being in a private school (aOR 1.43; 95% CI 1.20–1.71), consuming breakfast on < 3 days per week (aOR 1.47; 95% CI 1.22–1.76), consuming vegetables on < 3 days per week (aOR 1.29; 95% CI 1.03–1.59) and consuming sugar-sweetened drinks on < 3 days per week (aOR 1.42; 95% CI 1.16–1.75), whereas vigorous activity is associated with reduced risk of WHtR > 0.50 (medium: aOR 0.69, 95% CI 0.51–0.94; high: aOR 0.63, 95% CI 0.45–0.89).

**Table 4:** Associations between selected lifestyle factors and overweight/obesity and waist to height ratio (WHtR) in Saudi adolescents

**Model**	**Overweight/obesity**^**a**^				**WHtR**^**b**^			
	***β***	**SEE**	***p***	**aOR (95% CI)**	***β***	**SEE**	***p***	**aOR (95% CI)**
Intercept	- 0.128	0.620	0.836	-	−1.891	0.643	**0.003**	-
Age	- 0.036	0.037	0.341	0.97 (0.90-1.04)	0.036	0.039	0.344	1.04 (0.96-1.12)
Sex: Female ^**c**^	-	-	-	1.00	-	-	-	1.00
Male	0.547	0.092	**0.000**	1.73 (1.44-2.07)	0.421	0.095	**0.000**	1.52 (1.26-1.84)
School: Public ^**c**^	-	-	-	1.00	-	-	-	1.00
Private	0.402	0.088	**0.000**	1.50 (1.26-1.78)	0.360	0.091	**0.000**	1.43 (1.20-1.71)
Total physical activity: Low ^**c**^	-	-	-	1.00	-	-	-	1.00
Medium	- 0.238	0.243	0.327	0.79 (0.49-1.27)	- 0.106	0.278	0.702	0.89 (0.52-1.55)
High	- 0.357	0.271	0.187	0.69 (0.41-1.19)	- 0.180	0.515	0.473	0.83 (0.51-1.37)
Vigorous physical activity: low ^**c**^	-	-	-	1.00	-	-	-	1.00
Medium	- 0.334	0.148	**0.024**	0.72 (0.54-0.96)	- 0.366	0.152	**0.016**	0.69 (0.51-0.94)
High	- 0.413	0.170	**0.015**	0.66 (0.47-0.92)	- 0.463	0.175	**0.008**	0.63 (0.45-0.89)
Screen time: ≤ 2 hours/day ^**c**^	-	-	-	1.00	-	-	-	1.00
> 2–5 hours/day	0.086	0.128	0.500	1.09 (0.85-1.39)	0.002	0.132	0.987	1.00 (0.77-1.29)
> 5 hours/day	0.109	0.093	0.239	1.12 (0.93-1.34)	0.014	0.096	0.880	1.02 (0.84-1.22)
Breakfast intake: ≥ 5 days/week ^**c**^	-	-	-	1.00	-	-	-	1.00
3-4 days/week	0.107	0.131	0.415	1.11 (0.86-1.44)	0.074	0.137	0.591	1.08 (0.82-1.41)
< 3 days/week	0.361	0.090	**0.000**	1.44 (1.20-1.71)	0.382	0.093	**0.000**	1.47 (1.22-1.76)
Vegetables intake: ≥ 5 days/week ^**c**^	-	-	-	1.00	-	-	-	1.00
3-4 days/week	- 0.007	0.108	0.950	0.99 (0.80-1.23)	0.006	0.110	0.958	1.01 (0.81-1.25)
< 3 days/week	0.100	0.106	0.343	1.11 (0.90-1.36)	0.251	0.111	**0.024**	1.29 (1.03-1.59)
Fruits intake: ≥ 5 days/week ^**c**^	-	-	-	1.00	-	-	-	1.00
3-4 days/week	0.157	0.119	0.187	1.17 (0.93-1.48)	0.027	0.124	0.826	1.03 (0.81-1.31)
< 3 days/week	0.184	0.112	0.100	1.20 (0.97-1.49)	0.125	0.117	0.286	1.13 (0.90-1.43)
Milk intake: ≥ 5 d/week ^**c**^	-	-	-	1.00	-	-	-	1.00
3-4 days/week	0.011	0.111	0.920	1.01 (0.81-1.26)	0.061	0.115	0.599	1.06 (0.85-1.33)
< 3 days/week	0.176	0.099	0.075	1.19 (0.98-1.45)	0.199	0.102	0.051	1.22 (0.99-1.49)
Sugar-sweetened drink intake: ≥ 5 days/week ^**c**^	-	-	-	1.00	-	-	-	1.00
3-4 days/week	0.236	0.097	**0.015**	1.27 (1.05-1.53)	0.161	0.101	0.110	1.18 (0.96-1.43)
< 3 days/week	0.279	0.102	**0.006**	1.32 (1.08-1.62)	0.353	0.105	**0.001**	1.42 (1.16-1.75)

Data were analyzed by multivariate logistic regression adjusted for age.

^**a**^ Normal weight was used as the reference category.

^**b**^ WHtR < 0.5 was used as the reference category.

^**c**^ Reference category.

aOR = age-adjusted odds ratio; CI = confidence interval.

## Discussion

In the present study, we examined the associations between several lifestyle factors and overweight/obesity or abdominal obesity in Saudi adolescents aged 14–19 years, who were randomly selected from three major cities in Saudi Arabia. The main findings of this study are that males (compared with females) and adolescents in private (compared with public) schools have higher odds of being overweight or obese. In addition, logistic regression analysis indicated that Saudi adolescents had higher odds of being overweight/obese or abdominally obese if they less frequently engaged in vigorous physical activity, consumed breakfast or vegetables on < 3 days per week, and consumed sugar-sweetened beverages for 3–4 days per week or < 3 days per week.

The recommendations for the prevention and management of childhood obesity emphasize lifestyle modification, including daily moderate to vigorous physical activity, reducing television viewing and computer use, and avoiding unhealthy dietary habits, such as frequent consumption of fast foods, sugar-sweetened beverages, skipping breakfast and infrequent consumption of fruits and vegetables [10].

The current study showed that male adolescents had higher odds to be obese compared with their female peers. It is worth noting that the combined prevalence of overweight and obesity in many local and regional studies is higher in males than in females [2]. This is similar to the combined overweight and obesity profile of males and females among white American adolescents but opposite to that in black American adolescents [41]. However, the sex differences in rates of obesity among adolescents are generally small and inconsistent [42].

The findings of this study indicate that adolescents in private (compared with public) schools have higher odds of being overweight or obese. Adolescents in private schools usually come from families with higher socioeconomic status. At private schools, they may also have less restriction on food and snack choices compared with those in public schools. It is worth noting that studies on socioeconomic status and obesity suggest that the rate of obesity is higher among low income groups in developed countries, and in high income groups in developing countries [1,43]. This notion is supported by the present findings.

The finding that overweight/obesity was associated with lower levels of physical activity highlights the important role that physical activity, particularly vigorous activity, plays in preventing adolescent obesity. The present findings are consistent with the growing evidence showing that physical inactivity is a leading factor in obesity during childhood and adolescence [22,23,44-46]. In addition, insufficient vigorous physical activity was shown to be a risk factor for higher BMI in adolescent boys and girls in the United States [47]. Findings from a cross-sectional survey of adolescents aged 10–16 years from 34 countries demonstrated that physical activity levels were lower and television viewing times were higher in overweight compared to normal weight individuals [44]. An earlier study in Saudi Arabia [31] also showed that inadequate physical activity was associated with obesity in adolescents (OR 1.6; 95% CI 1.01–2.62). Similarly, a lack of exercise was a significant risk factor for obesity among adolescents from southwestern Saudi Arabia (aOR 1.35; 95% CI 1.06–1.94) [29]. Our finding that vigorous-intensity physical activity was inversely associated with adolescent obesity is also extensively supported by the literature. For example, Patrick et al. [47] studied a group of adolescents aged 11–15 years from the United States and found a significant association between overweight and vigorous-intensity physical activity but not with moderate-intensity physical activity. Furthermore, compared with moderate-intensity physical activity, a stronger negative association was reported between vigorous-intensity physical activity and total and central body fat in Spanish adolescents [48]. In addition, a review of the influence of physical activity on adiposity among 5–18 year olds concluded that a reduction in adiposity and an increase in aerobic capacity were observed when more time was spent on performing vigorous-intensity physical activity [49].

Excessive screen viewing in adolescence appears to be related to an unfavorable cardiovascular risk profile [50]. It is also believed that sedentary behaviors are associated with adverse health outcomes in a way that seems to be different from those attributed to the lack of physical activity [51]. In the current study, total screen viewing time was not associated with overweight or obesity. This finding is similar to those of several earlier studies that reported very weak [27] or no associations between screen viewing time and obesity [52]. However, other studies have reported significant associations between screen viewing time and obesity in children and adolescents [53-55]. In fact, some studies concluded that sedentary behaviors, such as television viewing, may be more important predictors of obesity indices in children than are physical activity behaviors [21]. However, ethnic and cultural factors may, at least partly, influence the associations between screen viewing time and obesity [53].

Some studies have suggested that there is an interaction effect between screen viewing time and physical activity that may influence the associations between obesity and sedentary behaviors. For example, Eisenman et al. [56] studied the combined effects of physical activity and television viewing on the risk of overweight and found that girls with high television viewing time and low physical activity had the highest odds of being overweight (OR 3.11). Meanwhile Dupuy et al. [23] reported that, in French adolescents aged 11–15 years who engaged in ≥ 60 min of moderate to vigorous physical activity on ≥ 5 days per week, television viewing was not associated with overweight, implying that high levels of physical activity may compensate for the negative effects of TV viewing. In the current study, we did not find a similar phenomenon nor was there an interaction between sedentary behavior and physical activity relative to obesity status. Furthermore, another recent study has shown that television viewing (sedentary activity) and physical activity appear to be separate entities that are independently associated with metabolic risk [54].

It appears that not all screen-based activities have equivalent associations with childhood obesity. Indeed, video games and computer use do not confer such high risk for obesity when compared with television viewing [57]. Research conducted on Australian adolescents has shown that computer use and video games were not significant risk factors for overweight or obesity [27]. However, in Brazilian adolescents, overweight was associated with more computer use [20]. In the present study, we did not find any significant differences between television viewing time or computer/video games use in relation to overweight or obesity; therefore, we combined both behaviors into a single variable.

Among all of the dietary habits assessed in the present study, overweight and obesity status was significantly associated with less frequent consumption of breakfast and sugar-sweetened drinks. In addition, reduced intake of vegetables was associated with increased odds of abdominal obesity. Other than the association with less frequent intake of sugar-sweetened drinks, our findings agree with those of many other studies. Indeed, skipping breakfast is a strong predictor of overweight and obesity in children and adolescents from many countries [15,20,58,59]. However, among 11–16-year-old Canadians, there was no clear association between dietary habits and measures of overweight and obesity [55]. The World Health Organization’s Global Strategy for Diet and Physical Activity recommend limiting energy intake from fats, reducing the intake of free sugars, and increasing fruit and vegetable consumption [60].

Surprisingly, the present study found significant inverse associations between the frequency of consuming sugar-sweetened drinks and measures of overall and abdominal obesity. This finding differs from those of many other studies. In a multivariate regression model, waist circumference was positively correlated with self-reported sugar-sweetened beverage intake among adolescent males [28]. Moreover, the consumption of sugar-sweetened drinks was found to be associated with obesity in children [61]. In a longitudinal study of non-obese adolescent girls, soda beverages were the only energy-dense snack food to be significantly associated with BMI z-scores over the 10-year study [62]. It has been suggested that sugar-sweetened beverages in liquid form could lead to increased caloric intake because they are less satiating than solid foods containing a similar energy content [61,62]. Our finding of the lower intake of sugar-sweetened beverages in overweight/obese subjects is difficult to interpret. It is possible, however, that the overweight/obese adolescents underestimated their intake of sugar-sweetened beverages and sweets. Another explanation is that overweight/obese adolescents might have been dieting and were switching to reduced-calorie beverages. In a 5-year longitudinal study, Vanselow et al. [26] found no association between sweetened beverage consumption and adolescent weight gain during the 5-year study. However, they did observe a positive association between low-caloric soft drinks and weight gain [26]. In our study, we did not assess the intake of reduced-calorie beverages.

A recent systematic review on the role of fruit and vegetable intake and obesity in children and adults concluded that increased fruit and vegetable consumption contributed to reduced adiposity among overweight or obese adults, but not among children, in experimental studies [63]. On the other hand, longitudinal studies of overweight adults showed marked associations between increased consumption of fruits and vegetables, and slower weight gain, but only one half of the children longitudinal studies indicated significant inverse associations between fruit and vegetable intake and obesity [63]. In the current study, we found a significant association between reduced vegetable, but not fruit, intake and increased odds of having abdominal obesity. The mechanism by which fruit and vegetable intake may prevent obesity is not fully understood, but could be related to the clustering effect of healthy habits. In our study, vegetable intake was correlated with frequency of breakfast consumption (*r* = 0.24, *p* < 0.01) and physical activity levels (*r* = 0.15, *p* < 0.01). In another study in Saudi adolescents, exercise was also positively correlated with fruit and vegetable intake [28].

Lifestyle-related factors, such as dietary habits, sedentary behavior and physical activity, all play an important role in creating an obesogenic environment. In Saudi Arabia, as well as other countries in the Eastern Mediterranean region, the pattern of food consumption pattern has changed enormously over the past four decades. During this time, the intake of animal products and refined sugar has increased while the intake of fruit and vegetables and complex carbohydrates has decreased [64]. In addition, Western calorie-dense fast foods are increasingly available and consumed by the young generation in this region. In the present study, there was no significant association between the frequency of fast food intake and obesity. In logistic regression models, fast food intake, along with several other variables, was highly correlated with other independent lifestyle factors, which were excluded from the logistic regression because they were considered redundant. However, the portion size of fast foods, which was not accounted for in this study, may be a confounding factor and could influence the association between the frequency of fast food consumption and obesity. Elsewhere, it was shown that fast food consumption markedly increased while frequency of skipping breakfast decreased during the transition from adolescence to adulthood, and both dietary behaviors were associated with weight gain during the same period [65]. Among Chinese children and adolescents, frequent consumption of snacks and Western fast food were associated with overweight and obesity [66].

The findings of the present study should be interpreted in light of its strengths and limitations. The main strength of our study is the large, representative and geographically diverse sample. We also used two indicators of obesity, namely cut-offs for BMI and WHtR. In addition, the physical activity questionnaire used in this study is valid and reliable. However, we must also acknowledge some of the limitations of the present study. The potential for recall bias in the frequency of physical activity, sedentary behaviors and dietary habits cannot be completely excluded. In addition, this was a cross-sectional study, which means we cannot infer causality from the current findings. In addition, the food frequency questionnaire did not account for portion size, which may influence the associations between dietary habits and measures of obesity.

## Conclusion

In conclusion, the present study examined the associations of several lifestyle factors with overweight/obesity and abdominal obesity in Saudi adolescents aged 14–19 years. Among all of the lifestyle factors assessed, overweight and obesity exhibited significant associations with less frequent vigorous physical activity, and less frequent consumption of breakfast, vegetables and sugar-sweetened beverages. Primary prevention of obesity by promoting a healthy diet and active lifestyles should be a national public health priority. Efforts designed to combat obesity among children and adolescents must include education, research and intervention, through the involvement of policy makers, health care providers, educators and parents.

## Competing interests

The authors declare that they have no competing interests.

## Authors’ contributions

**HMA** conceived the project and designed the research protocol, directed all aspects of this research, including supervising data collection in Riyadh, performed data analysis, and drafted the manuscript. **NAA** directed data collection in Al-Khobar and contributed to the writing of the manuscript. **HIA** was involved in training the female research assistants, supervised data collection in the female schools in Riyadh, and contributed to the writing of the manuscript. **DMQ** directed data collection for male participants in Jeddah, and contributed to the writing of the manuscript. **AOM** helped conceive the project and contributed to the writing of the manuscript. All authors critically read and approved the final version of the manuscript.

## Pre-publication history

The pre-publication history for this paper can be accessed here:

http://www.biomedcentral.com/1471-2458/12/354/prepub
